# Neutrophil-to-Lymphocyte Ratio (NLR)—Independent Prognostic Marker of Renal Function Decline in Chronic Kidney Disease: A Systematic Review and Meta-Analysis

**DOI:** 10.3390/jcm14196822

**Published:** 2025-09-26

**Authors:** Alexandru Burlacu, Calin Andrei Namolovan, Crischentian Brinza, Andreea Covic, Mariana Floria, Luminita Voroneanu, Adrian Covic

**Affiliations:** 1 Faculty of Medicine, University of Medicine and Pharmacy “Grigore T Popa”, 700115 Iasi, Romania; alexandru.burlacu@umfiasi.ro (A.B.); nca_13013@yahoo.com (C.A.N.); andreea.covic@gmail.com (A.C.); floria.mariana@umfiasi.ro (M.F.); lumivoro@yahoo.com (L.V.); adrian.covic@umfiasi.ro (A.C.); 2 Institute of Cardiovascular Diseases “Prof. Dr. George I.M. Georgescu”, 700503 Iasi, Romania; 3 Nephrology Clinic, Dialysis and Renal Transplant Center, “C.I. Parhon” University Hospital, 700503 Iasi, Romania; 4 Internal Medicine Clinic, “St. Spiridon” County Clinical Emergency Hospital Iasi, 700111 Iasi, Romania

**Keywords:** chronic kidney disease, end-stage kidney disease, estimated glomerular filtration rate, inflammation biomarkers, neutrophil-to-lymphocyte ratio

## Abstract

**Background/Objectives**: Chronic kidney disease (CKD) progression is strongly influenced by systemic inflammation. The neutrophil-to-lymphocyte ratio (NLR), derived from routine blood counts, reflects the balance between innate and adaptive immunity and has been proposed as a marker of adverse renal outcomes. We hypothesized that elevated baseline NLR is associated with reduced kidney function and increased risk of progression to end-stage kidney disease (ESKD) in adults with non-dialysis CKD. **Methods**: Following PRISMA guidelines, we systematically searched MEDLINE, Embase, and Scopus for studies enrolling adults with CKD stages 1–4 that reported renal outcomes according to baseline NLR. The primary outcome was progression to ESKD or renal replacement therapy (RRT) initiation. **Results**: Six eligible studies were identified, of which four provided sufficient data for meta-analysis. Across cohorts, elevated baseline NLR was consistently associated with adverse renal outcomes. In pooled analyses, high NLR nearly doubled the risk of ESKD or RRT (RR 1.96, 95% CI 1.30–2.97). Leave-one-out sensitivity analysis strengthened the association while reducing heterogeneity. For kidney function, higher NLR was associated with lower baseline eGFR (SMD −1.59, 95% CI −2.38 to −0.80). **Conclusions**: Elevated NLR is a robust prognostic marker of renal function decline and progression to ESKD or RRT in non-dialysis CKD. As a simple and inexpensive biomarker, NLR offers additional predictive value beyond eGFR and albuminuria. Incorporating NLR into risk models may refine stratification, guide follow-up, and enable earlier therapeutic optimization. Prospective large studies are warranted to establish thresholds and validate its role in clinical practice.

## 1. Introduction

Chronic kidney disease (CKD) is a growing global health burden, affecting approximately 10–16% of the population and increasing the risk of cardiovascular morbidity and progression to end-stage kidney disease (ESKD) [1,2]. A central mechanism underlying CKD progression is chronic low-grade systemic inflammation, which contributes to both glomerular damage and tubulointerstitial fibrosis through oxidative stress, cytokine activation, and immune dysregulation [2].

Among the biomarkers used to assess systemic inflammation, the neutrophil-to-lymphocyte ratio (NLR) has emerged as a simple, reproducible, and low-cost parameter that can be derived from standard complete blood counts [1,2,3,4,5]. NLR reflects the balance between neutrophil-mediated innate immunity and lymphocyte-mediated adaptive immunity, serving as an integrative index of inflammatory status. Compared to other markers such as high-sensitivity C-reactive protein (hs-CRP) or interleukin-6, NLR is more accessible and less susceptible to hepatic synthetic capacity or acute-phase fluctuations [1].

Several observational studies have explored the association between elevated NLR and adverse renal outcomes in CKD patients. Some have shown that NLR is an independent predictor of progression to dialysis or decline in renal function, particularly in patients with advanced CKD [1,2]. In contrast, others suggest that this association may vary by CKD stage or be confounded by comorbidities such as cardiovascular disease [6]. Still, conflicting findings exist—some studies failed to confirm a significant association after adjusting for eGFR, proteinuria, or inflammatory burden [7,8].

Given the increasing interest in using NLR for risk stratification in clinical nephrology and the growing body of evidence on its prognostic utility, a comprehensive synthesis is needed. The present systematic review aims to critically evaluate the current literature on the relationship between the NLR and renal outcomes—specifically, ESKD incidence and eGFR decline—in non-dialysis CKD patients (stages 1–4).

Through this review, we aim to determine whether NLR is an independent prognostic marker of CKD progression, whether its predictive value varies across CKD stages, and whether it adds clinical utility beyond established predictors, such as eGFR, albuminuria, and comorbid conditions [1,8,9].

## 2. Materials and Methods

The present systematic review was conducted in accordance with the updated guidelines of the Preferred Reporting Items for Systematic Reviews and Meta-Analyses (PRISMA) [10]. All methodological steps, including the literature search, study selection, data extraction, and synthesis, were predefined and followed.

### 2.1. Data Sources and Search Strategy

A comprehensive and systematic literature search was conducted in the following electronic databases: MEDLINE (PubMed), Embase, and Scopus, from 1 February 2025, to 30 May 2025. The search strategy combined free-text terms and controlled vocabulary (MeSH and Emtree terms) to ensure sensitivity and coverage of relevant studies. Search terms included variations and combinations of “neutrophil to lymphocyte ratio”, “NLR”, “chronic kidney disease”, “CKD”, “renal insufficiency”, “glomerular filtration rate”, “eGFR”, “end-stage kidney disease”, “end-stage renal disease”, “ESKD, and “kidney function”. No restrictions were applied regarding language, publication date, or geographic origin. In addition to database searches, reference lists of eligible articles and relevant reviews were screened to identify any additional studies. Moreover, the literature was also explored via Google Scholar and ClinicalTrials.gov. The complete search strategies for all databases are detailed in Supplementary Table S1.

### 2.2. Eligibility Criteria and Outcomes

Studies were selected based on predefined eligibility criteria formulated using the PICO framework (Table 1). We included prospective and retrospective studies that assessed the association between baseline neutrophil-to-lymphocyte ratio and adverse renal outcomes in adults with non-dialysis CKD. No restrictions were applied regarding the underlying etiology of CKD. Eligible studies enrolled participants aged 18 years or older and reported at least one relevant renal outcome.

The primary outcome was progression to end-stage kidney disease or initiation of chronic dialysis or kidney transplantation. The secondary outcome was estimated glomerular filtration rate (eGFR) according to NLR values. Studies were excluded if they involved patients already receiving dialysis or those with functioning renal transplants at baseline, or if they lacked stratification of outcomes by NLR categories. Case reports, reviews, conference abstracts, editorials, and studies without extractable outcome data were also excluded. In cases where multiple publications reported on overlapping cohorts, we retained only the most comprehensive or most recent study.

Two investigators independently screened all titles and abstracts retrieved from the database searches to assess eligibility. Full texts of potentially relevant studies were then reviewed to confirm inclusion. Any disagreements between reviewers were resolved through discussion and consensus.

### 2.3. Data Extraction and Synthesis

Two reviewers independently extracted data from eligible studies. The following variables were recorded: first author, year of publication, study design, sample size, patient characteristics (age, sex, CKD stage), baseline NLR levels or stratification (e.g., tertiles, quartiles), follow-up duration, reported outcomes, effect estimates (e.g., hazard ratios, odds ratios, or relative risks), and covariates included in the multivariable models. For studies reporting time-to-event outcomes, adjusted hazard ratios were preferentially extracted.

Due to anticipated heterogeneity across studies in terms of NLR cut-off values, outcome definitions, and adjustment covariates, a qualitative synthesis of findings was planned as the primary method of analysis. Nevertheless, when two or more studies reported comparable effect estimates and outcomes, we conducted a quantitative synthesis. Pooled estimates were calculated using a random-effects model. The degree of heterogeneity was assessed using the I^2^ statistic. Thresholds for I^2^ were interpreted as follows: low (<25%), moderate (25–50%), high (51–75%), and very high (>75%). Publication bias was assessed visually using funnel plot. All meta-analyses were performed using Review Manager (RevMan) version 5.4.1.

### 2.4. Quality Assessment

The methodological quality of each included study was independently assessed by two reviewers using the Newcastle-Ottawa Scale (NOS) for observational studies. This tool evaluates studies across three key domains: participant selection, comparability of study groups, and outcome assessment. Discrepancies in scoring were resolved by discussion or adjudication by a third reviewer.

## 3. Results

**Figure 1:**
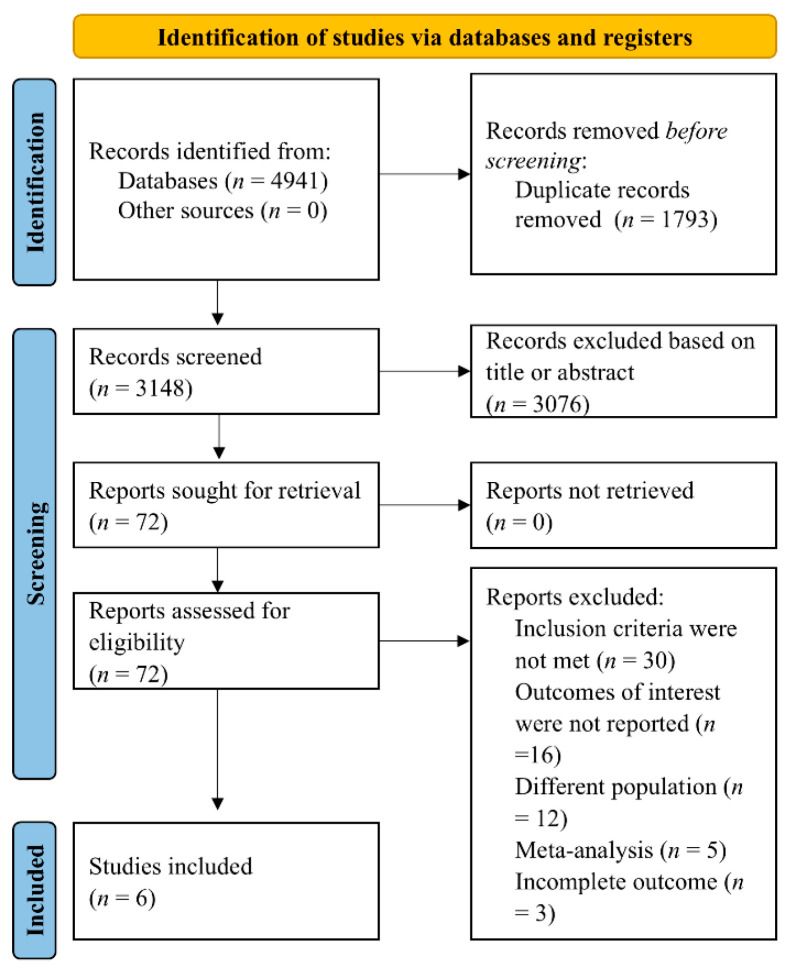
PRISMA flow chart of study selection in the present analysis.

The systematic search of prespecified databases yielded a total of 4941 records. After removal of duplicates and following title and abstract review, 72 full-text articles were assessed for eligibility. Ultimately, six studies were included in the systematic review, as described in the PRISMA flow chart (Figure 1).

The six cohorts were published between 2019 and 2023 and together enrolled adults with non-dialysis CKD stages 1–4 (Table 2) [1,2,6,7,8,9]. All studies were observational, and most used retrospective designs, while one employed a prospective [2]. Also, the analyzed studies included patients with follow-up ranging from approximately two to five and a half years. Baseline kidney function varied across cohorts. NLR was measured at baseline and analyzed using study-specific categories (e.g., tertiles, quartiles, or ROC-based cut-offs). Key design and population features are detailed in Table 2, and study-level outcomes in Table 3.

Across individual studies, a higher NLR was associated with worse kidney outcomes. In a large stage 2–4 CKD cohort, Altunoren et al. reported lower baseline eGFR and faster annual eGFR loss in the higher-NLR group. However, baseline NLR did not independently predict progression to stage 5 CKD or RRT after adjustment (Table 3) [6]. In IgA nephropathy, Chai et al. observed inferior renal survival in the highest NLR quartile and greater tubular atrophy/interstitial fibrosis on biopsy [7]. In another study, Kim et al. found that patients in the highest NLR tertile had substantially more composite kidney events (≥50% eGFR decline or RRT) [9]. In a larger series of IgA nephropathy, Wang et al. reported that a high NLR was associated with an increased risk of ESKD after multivariable adjustment [8]. In a prospective CKD cohort, Yoshitomi et al. recorded more composite events (dialysis or death) in the high-NLR group [2]. Additionally, Yuan et al. reported that a higher NLR was associated with incident ESKD, particularly in patients with CKD stage 4 [1].

(a) Progression to ESKD/RRT

**Figure 2:**
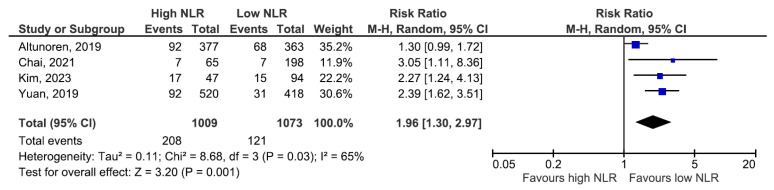
ESKD or RRT risk according to NLR values [1,6,7,9].

**Figure 3:**
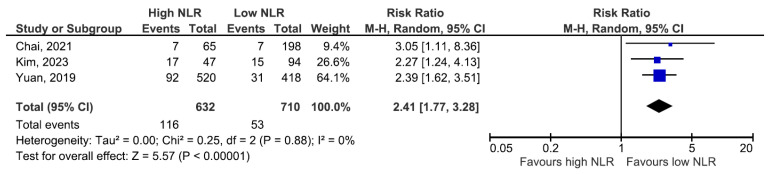
ESKD or RRT risk according to NLR values (low heterogeneity) [1,7,9].

In the pooled analysis, which included all studies that reported incident ESKD or RRT, the random-effects model showed a higher risk among patients with elevated baseline NLR (RR 1.96, 95% CI: 1.30–2.97), but with substantial between-study heterogeneity (I^2^ = 65%; Figure 2). We then performed prespecified sensitivity analyses. Using a leave-one-out approach to exclude the cohort contributing most to inconsistency yielded a larger pooled effect with low heterogeneity (RR 2.41, 95% CI: 1.77–3.28 (Figure 3)). The direction of association was concordant across cohorts despite differences in clinical context and NLR categorization. The heterogeneity observed among studies could reflect methodological differences, such as design and covariate adjustment, rather than representing conflicting directions of association.

(b) Baseline kidney function

**Figure 4:**
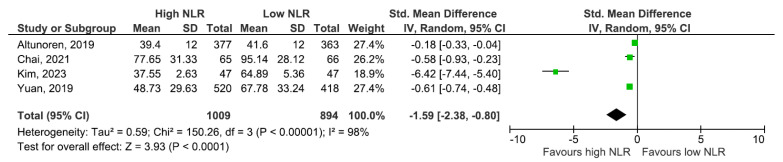
Baseline eGFR according to NLR values [1,6,7,9].

**Figure 5:**
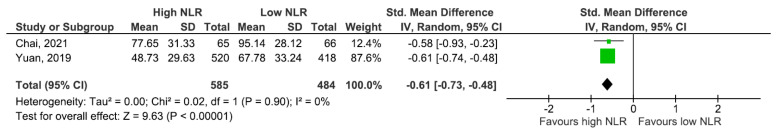
Baseline eGFR according to NLR values (low heterogeneity) [1,7].

In the all-study model comparing baseline renal function across NLR strata, a higher NLR was associated with a lower eGFR (SMD −1.59, 95% CI: −2.38 to −0.80), but with between-study heterogeneity of very high magnitude (I^2^ = 98%; Figure 4). Sensitivity analysis using a leave-one-out approach reduced heterogeneity, while the association persisted (SMD −0.61, 95% CI: −0.73 to −0.48 (Figure 5)). The large I^2^ in the complete model likely reflects structural differences across cohorts (stage distribution, study-specific NLR categorization) rather than a change in the direction of the effect.

**Figure 6:**
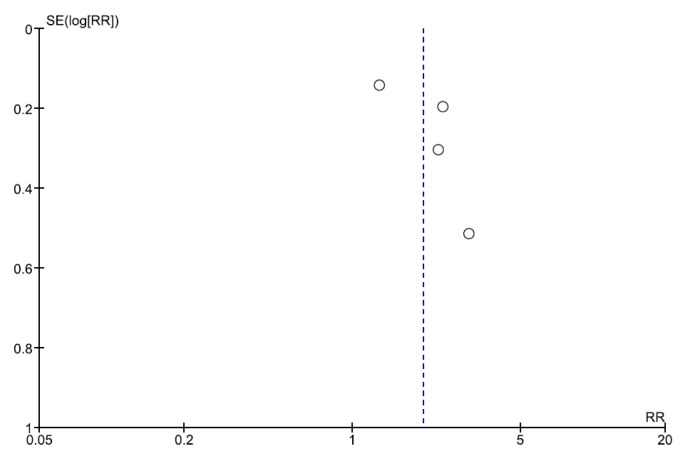
Funnel plot.

Also, the funnel plot appeared symmetric, with no evident small-study asymmetry (Figure 6). Quality appraisal with the Newcastle–Ottawa Scale classified all six studies as good, meeting predefined thresholds across selection, comparability, and outcome domains (Supplementary Table S2).

## 4. Discussion

This systematic review and meta-analysis emphasize that a higher NLR is associated with worse renal outcomes in adults with non-dialysis CKD. Across cohorts, elevated NLR was associated with lower baseline eGFR, and in pooled analyses, it was linked to a higher risk of progression to ESKD. The direction of effect was consistent, and sensitivity analyses, particularly leave-one-out methods, indicated that between-study variability was driven by study features rather than a reversal of the association.

In the pooled analysis, a higher NLR was associated with a nearly twofold increased risk of progression to ESKD or RRT. The direction of effect was aligned across studies. Importantly, in cohorts adjusted for established renal risk markers, including baseline eGFR and albuminuria, the association persisted, indicating supplementary prognostic information from NLR, not captured by these variables alone [1]. This meta-analysis demonstrates that NLR could serve as an independent risk prediction marker for progression to ESKD, supporting its consideration as an additive variable within current stratification frameworks.

NLR reflects an immune shift toward innate activation, with relative suppression of adaptive response. This pattern is typical of chronic, low-grade inflammation seen with aging, metabolic stress, endothelial dysfunction, and CKD. As a single, ratio-based index, NLR could provide a practical marker of this immune disequilibrium rather than serving as a nonspecific inflammatory surrogate [11,12,13]. Neutrophils in CKD exhibit pro-oxidant and proteolytic activity and can release neutrophil extracellular traps (NETs). These processes amplify endothelial injury, promote microvascular rarefaction, and contribute to cytokine signaling within the interstitium, thereby accelerating glomerular and tubular damage. These mechanisms connect NLR with endothelial dysfunction and atherosclerotic risk, especially in populations with overlapping diabetes, cardiovascular disease, and CKD. Moreover, a high NLR with lymphopenia reflects deficient adaptive immune control. CKD-related uremia, oxidative stress, and metabolic stressors impair the function of both effector and regulatory T cells, promoting inflammation and the activation of profibrotic pathways [12,13].

Compared with other inflammatory markers, NLR has practical advantages for routine CKD care. High-sensitivity CRP (hsCRP) and interleukin-6 (IL-6) are informative biomarkers that fluctuate with intercurrent comorbidities, adiposity, and hepatic synthetic capacity; however, they are not uniformly available as part of standard testing. By contrast, NLR is derived directly from the complete blood count, incurs no extra cost, and can be repeated at every visit, which facilitates longitudinal assessment [14]. The feasibility of NLR is also emphasized in disease-specific series, where it is highlighted as a convenient and repeatable index compared to more specialized tests [14].

Higher NLR has been linked to lower eGFR at baseline and to adverse clinical outcomes across CKD populations. In non-dialysis CKD, cohort studies showed that elevated NLR was associated with more advanced CKD and predicted adverse renal endpoints [9]. An earlier study in stage-4 CKD similarly reported that NLR predicted the transition to dialysis, reinforcing its relevance in advanced stages of CKD patients [15]. Beyond renal endpoints, data from a meta-analysis reported that NLR could predict all-cause mortality (HR 1.45, 95% CI: 1.20–1.75) and cardiovascular events (HR 1.52, 95% CI: 1.33–1.72) in CKD, supporting its role as a global risk marker in this population [16].

These data support integrating NLR as an adjunct, rather than a stand-alone test, alongside eGFR and albuminuria in risk stratification for patients with CKD. Patients with higher NLR could be prioritized for closer surveillance (more frequent monitoring of eGFR/albuminuria), earlier optimization of guideline-directed therapies, and consideration for multidisciplinary care. Therefore, incorporation of NLR into predictive tools (as an additional variable in kidney-failure risk models) is reasonable and could optimize risk stratification in this cohort of patients [6,8].

Most prior reports on NLR in CKD have been single-center cohorts with modest sample sizes and heterogeneous designs. By systematically assembling the available evidence and applying prespecified sensitivity analyses, this meta-analysis reconciles these discrepancies and demonstrates a consistent direction of effect across CKD populations and study designs. Beyond clarifying the literature, these findings establish a foundation for integrating NLR, measured from the routine complete blood count, into risk stratification frameworks alongside eGFR and albuminuria, and they define clear priorities for prospective validation and model development.

An important question is whether elevated NLR plays a causal role in kidney function decline, or whether it is mainly a consequence or indicator of underlying systemic inflammation, comorbidity, or decreased renal excretory capacity. The Chinese C-STRIDE study (stage 1–4 CKD) found that baseline higher NLR was associated with progression to ESKD in stage 4 CKD after multivariable adjustment, suggesting that declining kidney function itself may mediate or confound the relationship [1]. Mechanistic evidence supports that inflammation contributes to glomerular and tubulo-interstitial injury via oxidative stress, endothelial dysfunction and immune cell infiltration. Reactive oxygen species production and impaired antioxidant defenses could promote damage to endothelial cells and renal tubules in CKD [17]. Frąk et al. described the activation of NADPH oxidases and infiltration of neutrophils and macrophages in injured renal tissue, leading to fibrosis and glomerular injury [18]. However, given that many studies are observational, reverse causation remains a concern (i.e., lower eGFR may lead to accumulation of uremic toxins or oxidative mediators and altered immune cell counts) [19,20,21,22]. Therefore, while NLR is clearly a strong prognostic marker, further studies, preferably prospective with repeated measurements, are needed to delineate causality.

An important methodological consideration concerns the definition of high NLR, as no standardized threshold has been universally adopted in CKD research, and included studies have applied different approaches. For instance, some cohorts used median values (e.g., ≥1.9 or ≥2.7) to dichotomize patients, others relied on upper tertiles or quartiles (with values in the range of ~3.0–3.3), while several studies derived cut-off points using ROC analyses tailored to their population. As a result, the cut-off used to define high NLR varied considerably across studies, typically spanning values from approximately 1.9 to 3.3. This heterogeneity underlines the need for future research to establish standardized thresholds, which could enhance comparability across cohorts and facilitate the clinical application of NLR as a prognostic marker [1,2,6,7,9].

Moreover, recent evidence highlighted the prognostic role of NLR in mortality and cardiovascular complications among CKD patients. In an updated systematic review and meta-analysis including more than 26,000 individuals, Xu et al. reported that elevated NLR was associated with an increased risk of all-cause mortality (OR 1.22, 95% CI 1.15–1.29), major adverse cardiovascular events (OR 1.42, 95% CI 1.14–1.77), and cardiovascular mortality (OR 1.21, 95% CI 1.09–1.35) [23]. These findings extend the utility of NLR as a global prognostic marker in CKD, supporting its role in identifying patients at higher risk of adverse clinical outcomes [23].

Nevertheless, several limitations should be acknowledged. First, between-study heterogeneity reflected variability in NLR categorization (tertiles, quartiles, ROC-based thresholds), outcome definitions (pure ESKD vs. composite endpoints), and different CKD populations. Second, most cohorts were retrospective and observational, making them susceptible to residual confounding, despite the use of multivariable models, as acute intercurrent illnesses, corticosteroid exposure, or unmeasured inflammatory conditions may also have influenced leukocyte counts. Additionally, the limited number of eligible studies restricts formal exploration of publication bias and reduces power for subgroup analyses, including stage-specific thresholds. Notwithstanding these constraints, the direction of association was uniform across designs and settings, and sensitivity analyses that addressed influential cohorts confirmed the effect, supporting the overall inference that higher NLR is associated with worse renal outcomes in non-dialysis CKD.

Looking forward, NLR is a potential candidate variable for integration into composite risk tools alongside eGFR, albuminuria, age, and comorbidity. Future work should focus on external validation of such models across diverse healthcare settings, as well as their implementation into electronic health records to enable risk-aligned follow-up strategies. Moreover, cost-effectiveness analyses and interventional studies evaluating whether anti-inflammatory strategies or care pathways informed by NLR can improve clinical outcomes will be crucial to guide its adoption in routine practice.

## 5. Conclusions

In the present meta-analysis, an elevated NLR was consistently associated with lower baseline eGFR and a higher likelihood of progression to end-stage kidney disease in adults with non-dialysis CKD. The direction of effect was stable across cohorts and remained after sensitivity analyses. Given that NLR is derived from the complete blood count, requires no additional cost, and is easily repeated, it can now be used as an adjunct, rather than a stand-alone test, to refine risk stratification, prioritize closer surveillance, and support earlier optimization of guideline-directed care. Standardization of thresholds, use of serial measurements, and prospective multicenter validation, ideally via incorporation of NLR into established kidney-failure risk tools, are required to define its role in routine practice and to support guideline adoption.

## Figures and Tables

**Table 1:** Eligibility criteria based on PICO criteria.

Research Question	Is an elevated neutrophil-to-lymphocyte ratio (NLR) associated with increased risk of adverse renal outcomes, including progression to ESRD and an eGFR decline, in adults with CKD stages 1–4?	
Structured research question (PICO Format)	Population (P)	Adults with chronic kidney disease (CKD) stages 1–4, not on dialysis.
	Intervention/Exposure (I)	Elevated neutrophil-to-lymphocyte ratio (NLR), measured at baseline.
	Comparator (C)	Lower neutrophil-to-lymphocyte ratio (NLR), or within-study comparison groups stratified by NLR tertiles/quartiles.
	Outcomes (O)	Primary: progression to end-stage kidney disease (ESRD) or renal replacement therapy (RRT) initiation. Secondary: estimated glomerular filtration rate (eGFR).

**Table 2:** Summary of outcomes reported in the included studies.

Author, Year	Design	Patients, No	Age, Years	eGFR, mL/min/1.73 m^2^	Setting	Outcomes	Follow-Up
Altunoren, 2019 [6]	Observational, retrospective	740	62.8 ± 0.57	40.56 ± 0.45	Patients with CKD stage 2–4	Progression to stage 5 CKD or start of RRT	51.2 ± 30 months
Chai, 2021 [7]	Observational, retrospective	263	41.15 ± 12.64	86.68 ± 29.44	Patients with IgAN	(a) Renal tubular atrophy or interstitial fibrosis (b) Progression to ESKD	2 years
Kim, 2023 [9]	Observational, retrospective	141	56.47 ± 10.35	49.40 ± 29.76	Patients with non-dialysis CKD	(a) ≥50% eGFR decline or initiation of RRT (b) all-cause mortality	5.45 ± 2.11 years
Wang, 2021 [8]	Observational, retrospective	966	35	Median 96.23 (low NLR) and 87.05 (high NLR)	Patients with biopsy-proven IgAN	Progression to ESKD or start of RRT	58.67 months
Yoshitomi, 2019 [2]	Observational, prospective	350	68 (55–77)	33.6 (22.6–56.8)	Consecutive patients with CKD stage 1–4	ESKD requiring dialysis or death	31.8 months
Yuan, 2019 [1]	Observational, retrospective	938	52.8 ± 14.14	57.22 ± 32.67	Patients with CKD stage 1–4	(a) ESKD requiring RRT (b) CV events (c) all-cause death	4.55 years

CKD—chronic kidney disease; eGFR—estimated glomerular filtration rate; ESKD—end stage kidney disease; IgAN—IgA nephropathy; NLR—neutrophil-to-lymphocyte ratio; RRT—renal replacement therapy.

**Table 3:** Main outcomes and results of the included studies.

Author, Year	Outcomes	Results	*p*-Value
Altunoren, 2019 [6]	Progression to stage 5 CKD or start of RRT	Patients with high NLR had lower eGFR values (39.4 ± 12.0 vs. 41.6 ± 12.0 mL/min/1.73 m^2^)	*p* = 0.03
		High NLR was associated with increased annual eGFR decline (−4.96 ± 8.13 vs. −3.59 ± 6.15 mL/min/1.73 m^2^/year)	*p* = 0.04
		Baseline NLR was similar between patients who reached the endpoint and those who did not (3.54 ± 2.85 vs. 3.14 ± 1.99)	*p* = 0.09
		Baseline NLR was higher in patients with faster eGFR decline compared to those without (3.41 ± 1.82 vs. 3.13 ± 2.34)	*p* = 0.01
Chai, 2021 [7]	Progression to ESKD	Over 2 years, 14 patients developed ESRD (7 in highest quartile, 1 in lowest; renal survival rate 87.04% vs. 98.11%)	*p* = 0.029
	Tubular atrophy/ interstitial fibrosis	Higher NLR was associated with a greater proportion of tubular atrophy/interstitial fibrosis (T1/T2: 40% in highest NLR quartile vs. 22.73% in lowest)	*p* = 0.033
		NLR was an independent predictor of tubular atrophy/interstitial fibrosis (β = 1.230, 95% CI 0.081–2.379)	*p* = 0.036
Kim, 2023 [9]	Composite renal outcome	Highest NLR tertile (T3) had more events than lowest (T1): 55.3% vs. 17.0%; adjusted HR 3.33 (95% CI 1.43–7.76)	*p* = 0.005
	≥50% eGFR decline	Highest NLR tertile vs. lowest tertile: 51.1% vs. 12.8%; adjusted HR 3.12 (95% CI 1.23–7.91)	*p* = 0.017
	Initiation of RRT	Highest NLR tertile vs. lowest tertile: 36.2% vs. 8.5%; adjusted HR 2.87 (95% CI 0.89–9.25)	*p* = 0.078
	All-cause mortality	Highest NLR tertile vs. lowest tertile: 10.6% vs. 4.3%	*p* = 0.213
Wang, 2021 [8]	ESKD or start of RRT	High NLR associated with greater ESKD risk in univariate analysis (HR 2.68, 95% CI 1.60–4.50, *p* < 0.001) and remained an independent risk factor after multivariate adjustment (HR 1.74, 95% CI 0.98–3.05)	*p* = 0.043
Yoshitomi, 2019 [2]	ESKD requiring dialysis or death	Composite endpoint occurred in 83 patients, 54 in high NLR group vs. 29 in low NLR group; adjusted HR 1.67 (95% CI 1.02–2.77)	
Yuan, 2019 [1]	ESKD requiring RRT	Higher NLR levels were associated with an increased incidence of ESKD events	*p* < 0.001
		In CKD stage 4 patients, baseline NLR remained an independent predictor of ESKD after multivariable adjustment, with an HR of 2.12 (95% CI 1.10–4.10) compared with lower NLR.	*p* = 0.025

CKD—chronic kidney disease; eGFR—estimated glomerular filtration rate; ESKD—end stage kidney disease; NLR—neutrophil-to-lymphocyte ratio; RRT—renal replacement therapy.

## Data Availability

Not applicable.

## Supplementary Materials

The following supporting information can be downloaded at https://www.mdpi.com/article/10.3390/jcm14196822/s1. Table S1: Databases and search strategy; Table S2: Quality assessment.

## Abbreviations

The following abbreviations are used in this manuscript:

CKD	Chronic kidney disease
CV	Cardiovascular
eGFR	Estimated glomerular filtration rate
ESKD	End-stage kidney disease
hs-CRP	High-sensitivity C-reactive protein
IgAN	IgA nephropathy
IL-6	Interleukin-6
NLR	Neutrophil-to-lymphocyte ratio
NOS	Newcastle–Ottawa Scale
PRISMA	Preferred Reporting Items for Systematic Reviews and Meta-Analyses
RRT	Renal replacement therapy
SMD	Standardized mean difference
