# Supplementary material for: Neutrophil-to-Lymphocyte Ratio (NLR)—Independent Prognostic Marker of Renal Function Decline in Chronic Kidney Disease: A Systematic Review and Meta-Analysis
Source: J Clin Med. 2025 Sep 26;14(19):6822. doi: 10.3390/jcm14196822 (PMC12524446; doi:10.3390/jcm14196822)
Supplement: Supplementary file 1 [file jcm-14-06822-s001.zip › Table S1 Databases and search strategy.pdf]

Table S1. Databases and search strategies used in present systematic review and meta-analysis.

Database	Coverage	Search run	Records
**MEDLINE**	1946 to present	("Neutrophil-to-Lymphocyte Ratio" OR NLR) AND ("Renal Insufficiency, Chronic"[Mesh] OR CKD OR "chronic kidney disease" OR "renal dysfunction" OR "kidney disease" OR "renal insufficiency") AND ("glomerular filtration rate" OR eGFR OR "kidney function" OR "renal progression" OR "end-stage kidney disease" OR ESKD OR ESRD OR "renal replacement therapy" OR dialysis OR transplantation)	331
		("Neutrophil-to-Lymphocyte Ratio" OR NLR) AND ("glomerular filtration rate" OR eGFR OR "kidney function" OR "renal progression" OR "end-stage kidney disease" OR ESKD OR ESRD)	661
			Total records = 992
**Embase**	1966 to present	("Neutrophil-to-Lymphocyte Ratio" OR NLR) AND (CKD OR "chronic kidney disease" OR "renal dysfunction" OR "kidney disease" OR "renal insufficiency") AND ("glomerular filtration rate" OR eGFR OR "kidney function" OR "renal progression" OR "end-stage kidney disease" OR ESKD OR ESRD OR "renal replacement therapy" OR dialysis OR transplantation)	1433
		("Neutrophil-to-Lymphocyte Ratio" OR NLR) AND ("glomerular filtration rate" OR eGFR OR "kidney function" OR "renal progression" OR "end-stage kidney disease" OR ESKD OR ESRD)	1106
			Total records = 2539
**Scopus**	From the inception till present	("Neutrophil-to-Lymphocyte Ratio" OR NLR) AND (CKD OR "chronic kidney disease" OR "renal dysfunction" OR "kidney disease" OR "renal insufficiency") AND ("glomerular filtration rate" OR eGFR OR "kidney function" OR "renal progression" OR "end-stage kidney disease" OR ESKD OR ESRD OR "renal replacement therapy" OR dialysis OR transplantation)	371
		("Neutrophil-to-Lymphocyte Ratio" OR NLR) AND ("glomerular filtration rate" OR eGFR OR "kidney function" OR "renal progression" OR "end-stage kidney disease" OR ESKD OR ESRD)	1039
			Total records = 1410
All databases: 4941 records			
