# Supplementary material for: Neutrophil-to-Lymphocyte Ratio (NLR)—Independent Prognostic Marker of Renal Function Decline in Chronic Kidney Disease: A Systematic Review and Meta-Analysis
Source: J Clin Med. 2025 Sep 26;14(19):6822. doi: 10.3390/jcm14196822 (PMC12524446; doi:10.3390/jcm14196822)
Supplement: Supplementary file 1 [file jcm-14-06822-s001.zip › Table S2 Quality assessment.pdf]

Table S2. Quality assessment of included studies using Newcastle-Ottawa scale.

Study	Representativeness of the exposed cohort	Selection of the non-exposed cohort	Ascertainment of exposure	Presence of outcome of interest at the start	Comparability of cohorts	Assessment of outcome	Follow-up long enough for outcomes to occur	Adequacy of follow-up	Total
Altunoren, 2019	*	*	*	*	*	*	*		7
Chai, 2021	*	*	*	*	*	*	*		7
Kim, 2023	*	*	*	*	**	*	*		8
Wang, 2021	*	*	*	*	**	*	*		8
Yoshitomi, 2019	*	*	*	*	*	*	*		7
Yuan, 2019	*	*	*	*	**	*	*		8

Good quality: 3 or 4 stars in selection domain AND 1 or 2 stars in comparability domain AND 2 or 3 stars in outcome/exposure domain. Fair quality: 2 stars in selection domain AND 1 or 2 stars in comparability domain AND 2 or 3 stars in outcome/exposure domain. Poor quality: 0 or 1 star in selection domain OR 0 stars in comparability domain OR 0 or 1 stars in outcome/exposure domain.
